# 4-Thiazolidinones in Heterocyclic Synthesis: Synthesis of Novel Enaminones, Azolopyrimidines and 2-Arylimino-5-arylidene-4-thiazolidinones

**DOI:** 10.3390/molecules17066362

**Published:** 2012-05-25

**Authors:** Haider Behbehani, Hamada Mohamed Ibrahim

**Affiliations:** 1 Chemistry Department, Faculty of Science, Kuwait University, P.O. Box 5969, Safat 13060, Kuwait; 2 Chemistry Department, Faculty of Science, Fayoum University, Fayoum 63514, A. R., Egypt; E-Mail: hamadaaldeb@yahoo.com

**Keywords:** 4-thiazolidinone, enaminones, arylidene malononitrile, dimethylformamide dimethylacetal, azolopyrimidine

## Abstract

The 4-thiazolidinones **3a–d** were used as a key intermediates for the synthesis of 2-arylimino-5-arylidene-4-thiazolidinones derivatives **7a–p** *via* nucleophilic addition reactions with the arylidene malononitrile. Moreover the 4-thiazolidinones **3a** and **3c** condensed with the DMF-DMA to form the corresponding enamines **8** and **9** depending on the reaction conditions. Otherwise the 4-thiazolidinone **3b** reacts regioselectively with DMF-DMA to afford the enaminones **10** and **11**, respectively. The latter reacts with many heterocyclic amines affording polyfunctionally substituted fused pyrimidine derivatives **13–18**. The enamine **8b** was also reacted with the 3-amino-1,2,4-triazole to afford the acyclic product **19**, which could not be further cyclized to the corresponding tricyclic system **20**. Moreover the 4-thiazolidinone **3c** reacted with the benzenediazonium chloride to afford the arylhydrazones **12**. The X-ray single crystal technique was employed in this study for structure elucidation and *Z/E* potential isomerism configuration determination. The X-ray crystallographic analyses of eight products could be obtained, thus establishing with certainty the structures proposed in this work.

## 1. Introduction

One of the main objectives of organic and medicinal chemistry is the design, synthesis and production of molecules having value as human therapeutic agents and the treatment of infectious diseases still remains an important and challenging problem because of a combination of factors, including emerging infectious diseases and the increasing number of multi-drug resistant microbial pathogens, of particular relevance for Gram positive bacteria [1,2]. On the other hand, a recent survey of novel small-molecule therapeutics revealed that the majority of them result from an analogue-based approach and that their market value represents two-thirds of all drug sales [3]. There are numerous biologically active molecules with five membered rings, containing two heteroatoms among which is the 4-thiazolidinone ring system which is a core structure in various synthetic compounds and an important scaffold known to be associated with several biological activities such as hypnotic activity [4,5], antitubercular [6], anticonvulsant [7,8], antibacterial [9,10], anticancer [11,12], antihistaminic [13,14], antifungal [15], anti-inflammatory [16], antiviral [17] and cardiovascular effects [18]. Consequently, the combination of the 4-thiazolidinone template with substituted pyran or fused azolopyrimidine moieties which are also known to having several biological activities [19,20,21,22,23] in one molecule can be considered as promising approach in drug-like molecules design which is the goal of our study.

## 2. Results and Discussion 

**Scheme 1:**
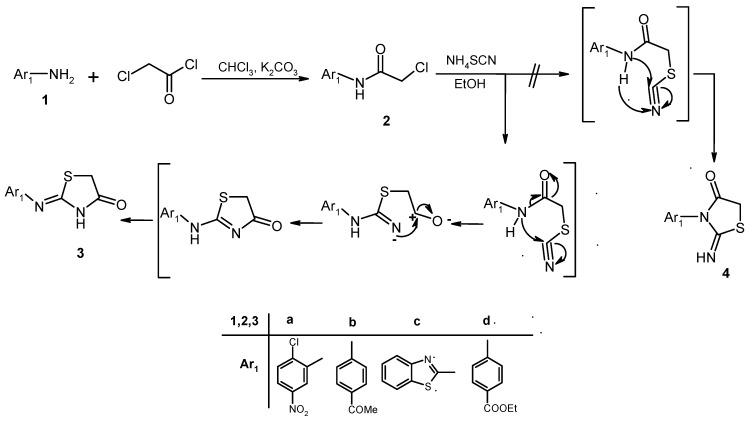
Synthesis of the 4-thiazolidinones **3a–d**.

**Figure 1:**
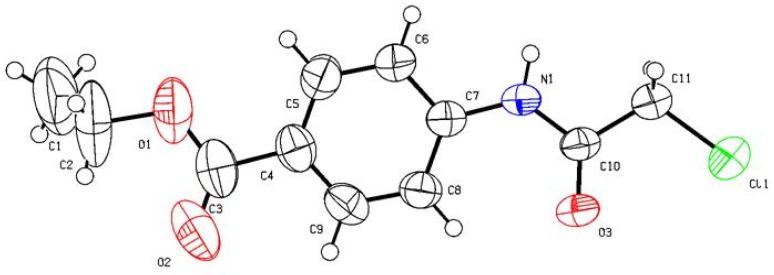
ORTEP plot of the X-ray crystallographic data determined for **2d**. Crystallo- graphic data have been deposited with the Cambridge Crystallographic Data Centre as supplementary publication number CCDC 874278 [28].

**Figure 2:**
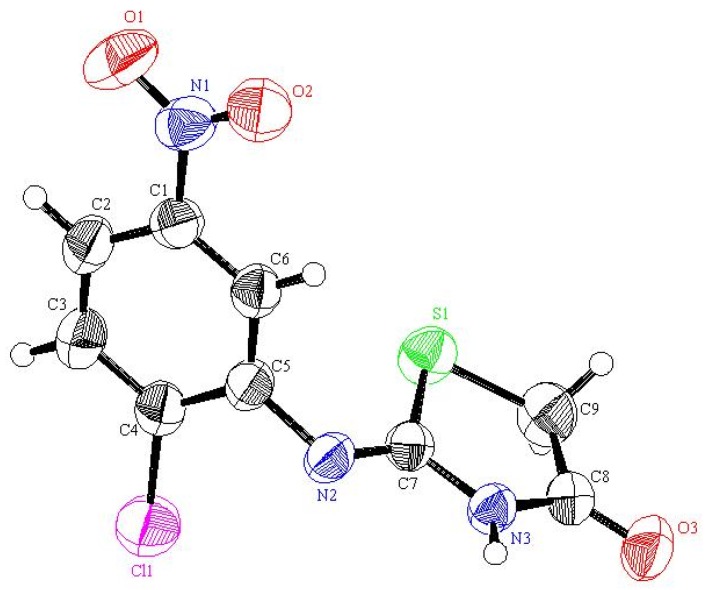
ORTEP plot of the X-ray crystallographic data determined for **3a**. Crystallo- graphic data have been deposited with the Cambridge Crystallographic Data Centre as supplementary publication number CCDC 874280 [29].

**Figure 3:**
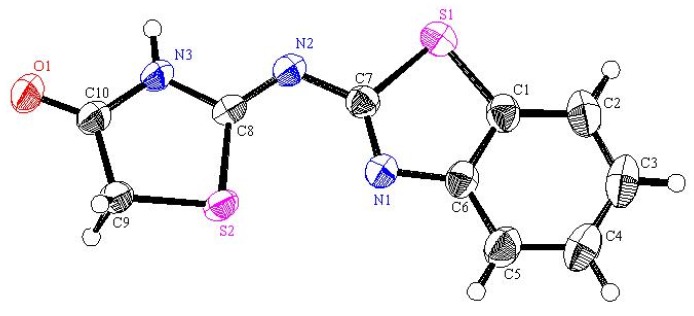
ORTEP plot of the X-ray crystallographic data determined for **3c**. Crystallo- graphic data have been deposited with the Cambridge Crystallographic Data Centre as supplementary publication number CCDC 874281 [30].

**Figure 4:**
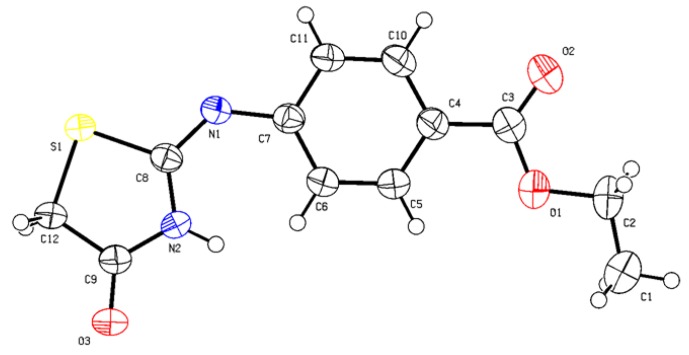
ORTEP plot of the X-ray crystallographic data determined for **3d**. Crystallo- graphic data have been deposited with the Cambridge Crystallographic Data Centre as supplementary publication number CCDC 874279 [31].

Several protocols for the synthesis of 4-thiazolidinones are available in the literature [24,25,26]. Here 2-chloro-*N*-(2-chloro-5-nitrophenyl)acetamide (**2a**), *N*-(4-acetylphenyl)-2-chloroacetamide (**2b**), 2-chloro-*N*-(benzothiazol-2-yl)acetamide (**2c**) and ethyl-4-(2-chloroacetamido)benzoate (**2d**) were synthesized using a procedure reported earlier [25] starting from 2-chloro-5-nitroaniline (**1a**), 4-amino acetophenone (**1b**), 2-aminobenzothiazole (**1c**) and benzocaine (**1d**), respectively, upon heterocyclization of **2a–d** in the presence of ammonium thiocyanate in refluxing ethanol, efficiently produced 2-(2-chloro-5-nitrophenyl-2-ylimino)thiazolidin-4-one (**3a**), 2-(4-acetylphenylimino)thiazol- idin-4-one (**3b**), 2-(benzothiazol-2-ylimino)thiazolidin-4-one (**3c**) and ethyl-4-(4-oxothiazolidin-2-ylideneamino)benzoate (**3d**), through intramolecular cyclization and the Dimroth-like rearrangements [27] (cf. Scheme 1). The lactam structure for **3a–d** was confirmed based on the ^1^H-NMR spectra which exhibit the NH proton at δ ≈ 12.35 ppm, accounting for a lactam proton but not for an imine proton which is expected around 9.0 ppm. The lactam structure was also confirmed through the X-ray single crystal structure determination for **3a**, **3c** and **3d** (cf. Figure 1, Figure 2, Figure 3, Figure 4 and Table 1, Table 2). This was considered to be a strong evidence for this type of ring closure which gives **3** and not **4** [25] as mentioned by Vicini, *et al.* [26].

Inspection of the crystallographically determined lengths of the N2-C7 bond (1.28 Å) in **3a**, N2-C8 bond (1.30 Å) in **3c** and N1-C8 bond (1.28 Å) in **3d** showed that they are typical C=N bond lengths.

**Scheme 2:**
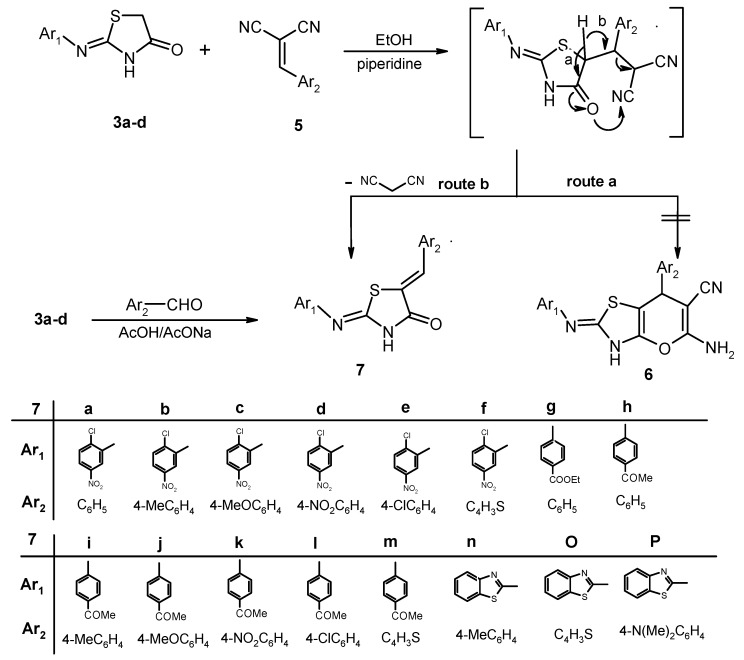
Synthesis of the 5-benzylidene-4-thiazolidinone derivatives **7**.

**Figure 5:**
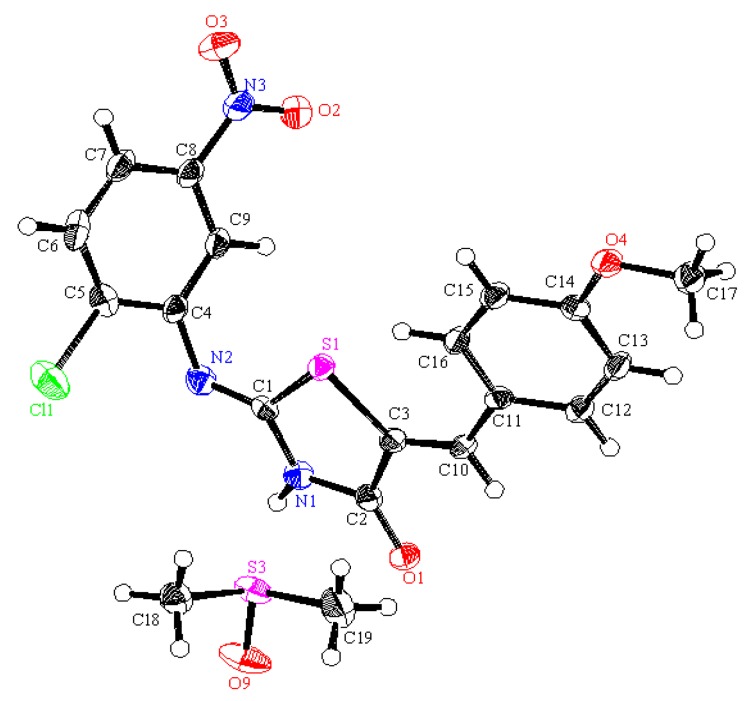
ORTEP plot of the X-ray crystallographic data determined for **7c** containing a DMSO molecule. Crystallographic data have been deposited with the Cambridge Crystallographic Data Centre as supplementary publication number CCDC 875772 [34].

It was of interest to explore the scope, limitations and generality of the 4-thiazolidinones **3** as a precursor for the synthesis of some polyfunctionally substituted fused pyran derivatives for which we might expect a wide spectrum of bioresponses. Thus the active methylene in the 4-thiazolidinones **3a-d** underwent nucleophilic addition reaction to the double bond of a variety of arylidene malononitriles **5** *via* a Michael type addition [32] reaction, by refluxing in ethanol containing few drops of piperidine to give a substance whose structure should be either the pyranothiazole derivative **6** (route a) or the arylidene derivatives **7** (route b). The actual structure of the product was assigned as 5-arylidene-4-thiazolidinone derivatives **7** based on the molecular mass of the product which in agreement with this structure, moreover the ^1^H-NMR lacked the pyran H-4 signal which should appear at approximately δ = 4.0–5.0 ppm [33]. Also the ^1^H-NMR spectroscopic data showed a NH signal and was devoid of an amino group signal which should have appeared if the reaction product were **6**. Compounds **7** were also obtained *via* refluxing of 4-thiazolidinones **3a–d** with the appropriate aldehydes in glacial acetic acid containing sodium acetate. The *Z* configuration of the products was confirmed *via* the X-ray crystallographic analysis (cf. Scheme 2 and Figure 5). Attempts to isolate the targeted pyranothiazole derivative **6** failed. The main advantage of method a over method b is that it gives a higher yield, as illustrated in the Experimental section.

**Scheme 3:**
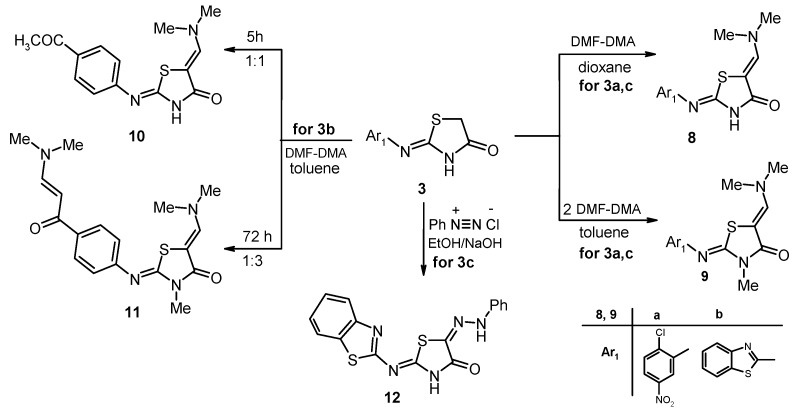
Reaction of **3a-c** with DMF-DMA and bezenediazonium chloride.

In order to synthesis a new class of enamine derivatives the active methylene in the 4-thiazol- idinones **3a** and **3c** was condensed with dimethylformamide dimethylacetal (DMF-DMA) in dioxane to yield the corresponding enamines **8a,b** (cf. Scheme 3). 

**Figure 6:**
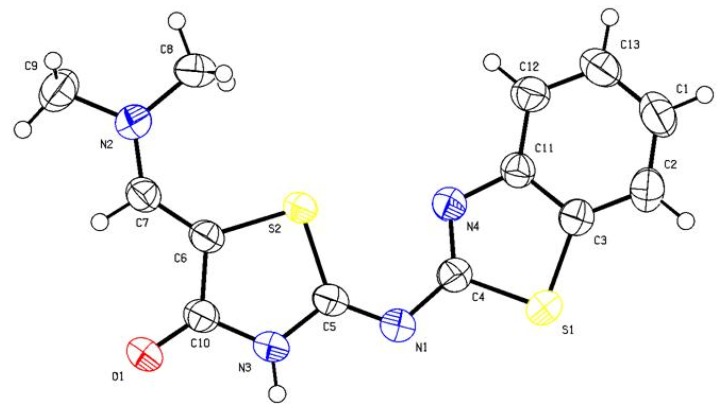
ORTEP plot of the X-ray crystallographic data determined for **8b**. Crystallo- graphic data have been deposited with the Cambridge Crystallographic Data Centre as supplementary publication number CCDC 874404 [35].

**Figure 7:**
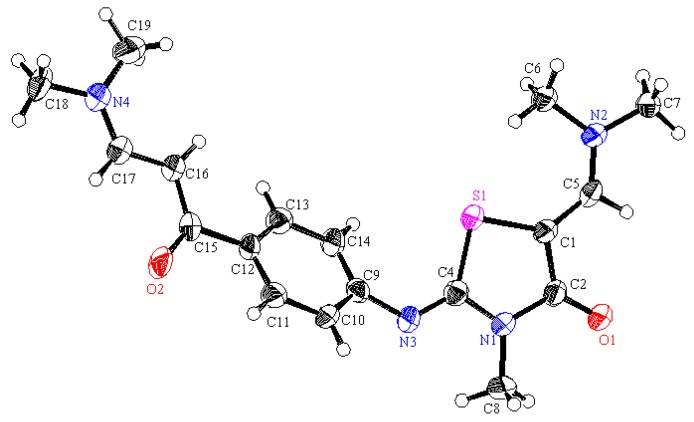
ORTEP plot of the X-ray crystallographic data determined for **11**. Crystallo- graphic data have been deposited with the Cambridge Crystallographic Data Centre as supplementary publication number CCDC 880545 [36].

The fact that only the *Z*-isomer could be formed was confirmed by X-ray single crystal determination (cf. Figure 6 and Table 3). The methylated enamines **9a,b** were also formed as products when the reaction was carried out in toluene in the presence of excess DMF-DMA. On the other hand the 4-thiazolidinones **3b** condensed with DMF-DMA regioselectively in toluene to afford the enaminones **10** and **11**, respectively, depending on the reaction conditions. The structure and configuration of the enaminone **11** was also determined *via* the X-ray single crystal technique (cf. Figure 7). Moreover the active methylene in **3c** was coupled with the benzenediazonium chloride in EtOH/NaOH to form the corresponding arylhydrazone **12** (cf. Scheme 3).

The foregoing results prompted us to investigate the behavior of the enaminone **11** towards some N-nucleophiles such as heterocyclic amines as potential precursors for polyfunctionally substituted fused pyrimidine derivatives which display a broad spectrum of biological activity [19,20,21,22,23].

**Scheme 4:**
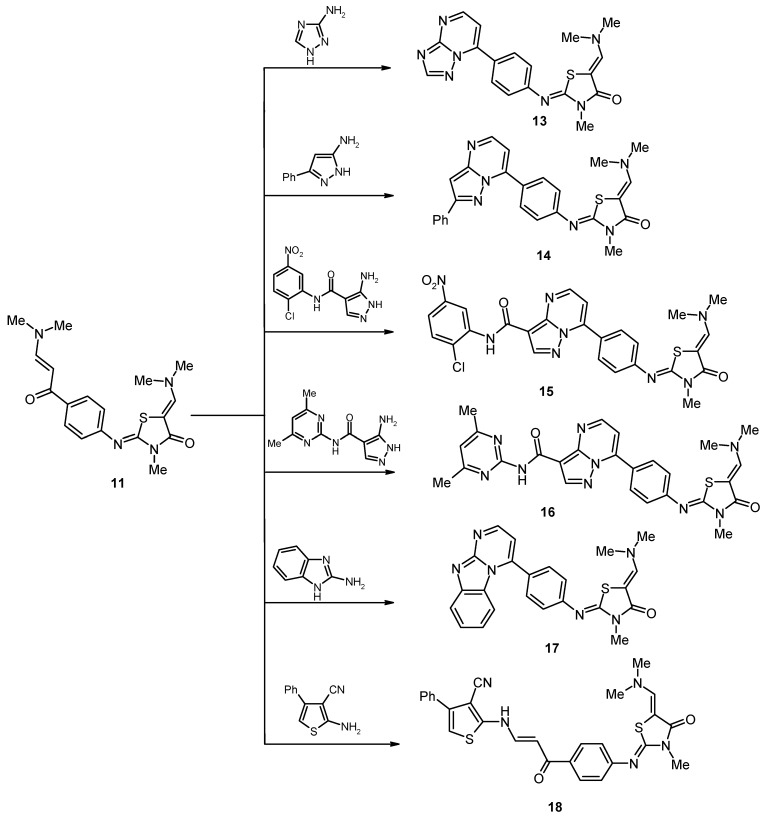
Reactions of the enaminone **11** with heterocyclic amines.

**Figure 8:**
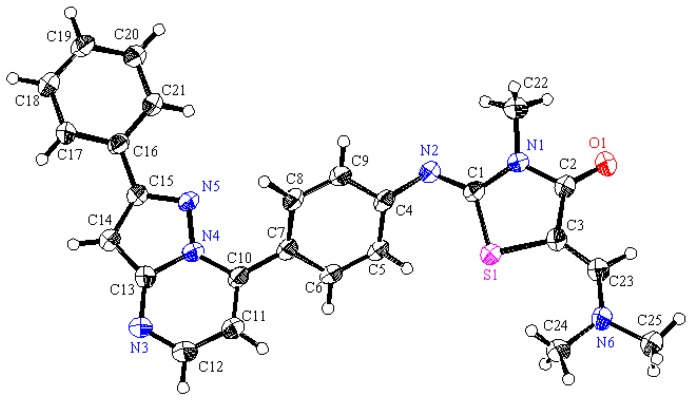
ORTEP plot of the X-ray crystallographic data determined for **14**. Crystallo- graphic data have been deposited with the Cambridge Crystallographic Data Centre as supplementary publication number CCDC 880544 [37].

Thus, reaction of the enaminone **11** with equimolar amounts of various heterocyclic amines, namely 3-amino-1,2,4-triazole, 3-phenyl-1*H*-pyrazole-5-amine, 5-amino-*N*-(2-chloro-5-nitrophenyl)-1*H*-pyrazole-4-carboxamide, 5-amino-*N*-(4,6-dimethylpyrimidin-2-yl)-1*H*-pyrazole-4-carboxamide upon reflux in pyridine, afforded the corresponding 1,2,4-triazolo[1,5-*a*]pyrimidine **(13**) and pyrazolo[1,5-*a*] pyrimidine derivatives **14–16** (cf. Scheme 4). The identity of the products was established on the basis of elemental analyses and spectral background in each case also, the X-ray single crystal technique was employed for this purpose and for determination of the configuration of the products (cf. Figure 8). In the same manner, the enaminone **11** reacts regioselectively with 2-aminobenzimidazole under the same experimental condition to give the benzimidazo[1,2-*a*]pyrimidine derivative **17**. The structure of compound **17** was established on the basis of elemental analysis and spectral data of the isolated reaction product. Formation of the fused pyrimidine derivatives **13–17** from the enaminone **11** and N,N-binucleophiles proceeds by initial substitution of the dimethylamino group followed by cyclization. Similarly, the reaction of the enaminone **11** with 2-amino-4-phenylthiophene-3-carbonitrile in refluxing pyridine furnished only one isolable product, which was identified as **18**. The spectral data of the isolated product were in complete agreement with the assigned structure. For example, the IR spectrum of the reaction product **18** revealed bands due to nitrile function, NH and two carbonyl at 2206, 3116, 1699, 1654 cm^−1^, respectively (cf. Scheme 4).

**Scheme 5:**
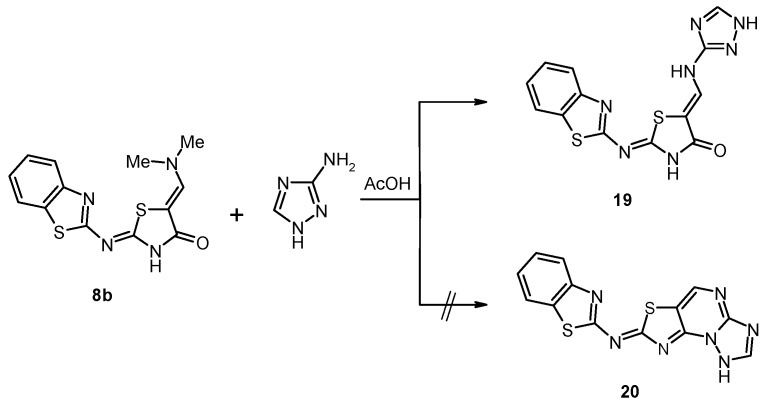
Reaction of **8b** with 3-amino-1,2,4-triazole.

In attempts to synthesis a novel class of fused azoloazinethiazolidinone derivatives **20** we reacted the enamine **8b** with 3-amino-1,2,4-triazole as an example of a heterocyclic amine but the formed product was found to be the acyclic one **19** and not the fused one **20**. Attempts to effect cyclisation of **19** into **20** under a variety of conditions failed (cf. Scheme 5).

## 3. Experimental

### 3.1. General 

Melting points were recorded on a Griffin melting point apparatus and are reported uncorrected. IR spectra were recorded using KBr disks using a Perkin-Elmer System 2000 FT-IR spectrophotometer. ^1^H-NMR (400 MHz) or (600 MHz) and ^13^C-NMR (100 MHz) or (150 MHz) spectra were recorded at 25 °C or as reported in CDCl_3_ or DMSO-*d_6_* as solvent with TMS as internal standard on a Bruker DPX 400 or 600 super-conducting NMR spectrometer. Chemical shifts are reported in ppm. Mass spectra were measured using a high resolution GC-MS (DFS) Thermo spectrometer with EI (70 EV). Microanalyses were performed on a LECO CHNS-932 Elemental Analyzer. The crystal structures were determined by a Rigaku R-AXIS RAPID diffractometer and Bruker X8 Prospector at Kuwait University. The compounds 5-amino-*N*-(2-chloro-5-nitrophenyl)-1*H*-pyrazole-4-carboxamide and 5-amino-*N*-(4,6-dimethylpyrimidin-2-yl)-1*H*-pyrazole-4-carboxamide were prepared according to the literature procedure [38]. 

### 3.2. General Procedure for the Preparation of 2-Chloro-N-(heteroaryl)acetamides ***2a–d***

A solution of the appropriate amines **1a–d** (10 mmol) and chloroacetyl chloride (1.12 g, 10 mmol) in chloroform (50 mL) was refluxed in the presence of K_2_CO_3_ (15 mmol) for about 10 h. Then the solvent was removed *in vacuo* and the residue was stirred with water (100 mL) and filtered. The solid product is then washed with 5% NaHCO_3_ solution and subsequently with water. The crude product is dried and crystallized from appropriate solvent to furnish pure solid product.

*2-Chloro-N-(2-chloro-5-nitrophenyl)acetamide* (**2a**). Recrystallized from isopropyl alcohol as pale yellow crystals, yield: 93%, m.p. 120–121 °C; IR (KBr): 𝑣/cm^−1^ 3354 (NH), 1686 (CO); ^1^H-NMR (DMSO-*d_6_*): δ = 4.47 (s, 2H, CH_2_), 7.84 (d, *J* = 8.0 Hz, 1H, Ar-H), 8.06 (d, *J* = 8.0 Hz, 1H, Ar-H), 8.73 (s, 1H, Ar-H), and 10.23 ppm (s, 1H, NH); ^13^C-NMR (DMSO-*d_6_*): δ = 43.03 (CH_2_), 115.96, 119.91, 129.44, 129.77, 134.58, 147.17 and 164.18 ppm (Ar-C and CO) MS (EI): *m*/*z* (%) 248 (M^+^, 11.55), 249 (M^+^+1, 3.75). Anal. calcd. for C_8_H_6_Cl_2_N_2_O_3_ (249.05): C, 38.58; H, 2.43; N, 11.25. Found: C, 38.66; H, 2.50; N, 11.18.

*N-(4-Acetylphenyl)-2-chloroacetamide* (**2b**). Recrystallized from EtOH as yellow crystals, yield: 89%, m.p. 145–146 °C; IR (KBr): 𝑣/cm^−1^ 3286 (NH), 1707, 1658 (2CO); ^1^H-NMR (DMSO-*d_6_*): δ = 2.53 (s, 3H, CH_3_), 4.31 (s, 2H, CH_2_), 7.73 (d, *J* = 8.4 Hz, 2H, Ar-H), 7.94 (d, *J* = 8.4 Hz, 2H, Ar-H) and 10.63 ppm (s, 1H, NH); *m*/*z* (%) 211 (M^+^, 39.20), 212 (M^+^+1, 7.65). Anal. calcd. for C_10_H_10_ClNO_2_ (211.65): C, 56.75; H, 4.76; N, 6.62. Found: C, 56.84; H, 4.72; N, 6.58.

*N-Benzothiazol-2-yl-2-chloroacetamide* (**2c**) [39]. Recrystallized from EtOH as white crystals, yield: 96%, m.p. 145–146 °C; IR (KBr): 𝑣/cm^−1^ 3348 (NH), 1665 (CO); ^1^H-NMR (DMSO-*d_6_*): δ = 4.47 (s, 2H, CH_2_), 7.32 (t, *J* = 7.6 Hz, 1H, Ar-H), 7.45 (t, *J* = 7.6 Hz, 1H, Ar-H), 7.77 (d, *J* = 7.6 Hz, 1H, Ar-H), 7.99 (d, *J* = 7.6 Hz, 1H, Ar-H) and 12.74 ppm (s, 1H, NH); ^13^C-NMR (DMSO-*d_6_*): δ = 43.02 (CH_2_), 121.16, 122.26, 124.26, 126.70, 131.94, 148.87, 158.06 and 166.43 ppm (Ar-C and CO); MS (EI): *m*/*z* (%) 226 (M^+^, 26.4), 227 (M^+^+1, 5.6). Anal. calcd. for C_9_H_7_ClN_2_OS (226.69): C, 47.69; H, 3.11; N, 12.36; S, 14.14. Found: C, 47.77; H, 3.09; N, 12.44; S, 14.23.

*Ethyl-4-(2-chloroacetamido)benzoate* (**2d**). Recrystallized from EtOH as white crystals, yield: 91%, m.p. 106–107 °C; IR (KBr): 𝑣/cm^−1^ 3275 (NH), 1722, 1678 (2CO); ^1^H-NMR (CDCl_3_): δ = 1.36 (t, *J* = 7.2 Hz, 3H, CH_3_), 4.17 (s, 2H, CH_2_), 4.34 (q, *J* = 7.2 Hz, 2H, CH_2_), 7.63 (d, *J* = 8.0 Hz, 2H, Ar-H), 8.01 (d, *J* = 8.0 Hz, 2H, Ar-H) and 8.56 ppm (s, 1H, NH); ^13^C-NMR (CDCl_3_): δ = 14.22 (CH_3_), 42.84 (CH_2_), 60.93 (CH_2_), 119.12, 126.74, 130.68, 140.75, 164.15 and 165.92 ppm (Ar-C and CO); MS (EI): *m*/*z* (%) 241 (M^+^, 68.84), 242 (M^+^+1, 21.72). Anal. calcd. for C_11_H_12_ClNO_3_ (241.68): C, 54.67; H, 5.00; N, 5.80. Found: 54.75; H, 4.93; N, 5.71. Crystallographic Analysis for **2d**: The crystals were mounted on a glass fiber. All measurements were performed on Bruker X8 Prospector. The data were collected at a temperature of 20 ± 1 °C to a maximum θ value of 66.61° using the ω scanning technique. The structure was solved by direct method using SHELXS-97 (Sheldrick, 2008) and refined by Full-matrix least-squares on F^2^. The non-hydrogen atoms were refined anisotropically. Data were corrected for absorption effects using the multi-scan method (SADABS). Crystal Data: C_11_H_12_ClNO_3_, M = 241.68, monoclinic, a = 4.7349(4) Å, b = 28.541(2) Å, c = 9.1098(6) Å, V = 1203.41(16) Å^3^, α = γ = 90.00°, β = 102.173(4)°, space group: P 1 21/n 1, Z = 4, D_calc_ = 1.334 Mg cm^−3^, No. of reflections measured 4589, θ_max_ = 66.34°, R1 = 0.06. Figure 1 illustrates the structure as determined. Full data can be obtained on request from the CCDC [28].

### 3.3. General Procedure for the Synthesis of 2-(Arylimino)thiazolidin-4-ones ***3a–d***

A solution of 2-chloro-*N*-(heteroaryl or aryl)acetamides **2a–d** (10 mmol) and ammonium thiocyanate (15 mmol) in absolute ethanol (30 mL) was refluxed for 4 h and allowed to stand overnight. The formed precipitate was filtered off, washed with water and then recrystallised from the appropriate solvent.

*(Z)-2-(2-Chloro-5-nitrophenylimino)thiazolidin-4-one* (**3a**). Recrystallized from EtOH as yellow crystals, yield: 76%, m.p. 198–199 °C; IR (KBr): 𝑣/cm^−1^ 3136 (NH), 1738 (CO); ^1^H-NMR (DMSO-*d_6_*): δ = 4.09 (s, 2H, CH_2_), 7.81 (d, *J* = 8.4 Hz, 1H, Ar-H), 7.89 (s, 1H, Ar-H), 7.98 (d, *J* = 8.4 Hz, 1H, Ar-H) and 12.23 ppm (s, 1H, NH); ^13^C-NMR (DMSO-*d_6_*): δ = 34.57 (CH_2_), 116.96, 119.99, 131.12, 133.19, 146.50, 146.78, 160.11 and 173.73 (Ar-C and CO); MS (EI): *m*/*z* (%) 271 (M^+^, 100), 272 (M^+^+1, 32.25). Anal. calcd. for C_9_H_6_ClN_3_O_3_S (271.68): C, 39.79; H, 2.23; N, 15.47; S, 11.80; Found: C, 39.86; H, 2.14; N, 15.36; S, 11.88. Crystallographic Analysis for **3a**: The crystals were mounted on a glass fiber. All measurements were performed on a Rigaku R-AXIS RAPID diffractometer using filtered Mo-Kα radiation. The data were collected at a temperature of 20 ± 1 °C to a maximum 2θ value of 55.0° using the ω scanning technique. The structure was solved by charge flipping method and expanded using Fourier techniques. The non-hydrogen atoms were refined anisotropically. Hydrogen atoms were refined using the riding model. Crystal Data: C_9_H_6_ClN_3_O_3_S, M = 271.68, orthorhombic, a = 20.1723(7) Å, b = 11.3976(5) Å, c = 20.145(2) Å, V = 4631.6(5) Å^3^, α = β = γ = 90.00°, space group: Pbca (#61), Z = 16, D_calc_ = 1.558 g cm^−3^, No. of reflections measured 5236, 2θ_max_ = 54.9°, R1 = 0.0683. Figure 2 shows the structure as determined. Full data can be obtained on request from the CCDC [29].

*(Z)-2-(4-Acetylphenylimino)thiazolidin-4-one* **(3b)**. Recrystallized from EtOH/dioxane (1:1) as buff crystals, yield: 81%, m.p. 260–261 °C; IR (KBr): 𝑣/cm^−1^ 3271 (NH), 1717, 1666 (2CO); ^1^H-NMR (DMSO-*d_6_*): δ = 2.55 (s, 3H, CH_3_), 4.03 (s, 2H, CH_2_), 7.06 (d, *J* = 8.0 Hz, 1H, Ar-H), 7.86 (d, *J* = 8.0 Hz, 1H, Ar-H), 7.96 (d, *J* = 8.0 Hz, 2H, Ar-H) and 11.92 ppm (s, 1H, NH); ^13^C-NMR (DMSO-*d_6_*): δ = 26.49 (CH_3_), 37.39 (CH_2_), 118.56, 129.57, 132.26, 142.57, 164.90, 174.01 and 196.49 (Ar-C and CO); MS (EI): *m*/*z* (%) 234 (M^+^, 78.0), 235 (M^+^+1, 15.80). Anal. calcd. for C_11_H_10_N_2_O_2_S (234.28): C, 56.40; H, 4.30; N, 11.96; S,13.69. Found: C, 56.33; H, 4.26; N, 11.85; S,13.55.

*(Z)-2-(Benzothiazol-2-ylimino)thiazolidin-4-one* (**3c**) [36]. Recrystallized from an EtOH/dioxane (1:1) mixture pale yellow crystals, yield: 73%, m.p. 201–202 °C; IR (KBr): 𝑣/cm^−1^ 3145 (NH), 1734 (CO); ^1^H-NMR (DMSO-*d_6_*): δ = 4.12 (s, 2H, CH_2_), 7.39 (t, *J* = 7.6 Hz, 1H, Ar-H), 7.51 (t, *J* = 7.6 Hz, 1H, Ar-H), 7.85 (d, *J* = 7.6 Hz, 1H, Ar-H), 8.01 (d, *J* = 7.6 Hz, 1H, Ar-H) and 12.35 ppm (s, 1H, NH); ^13^C-NMR (DMSO-*d_6_*): δ = 35.68 (CH_2_), 121.82, 122.40, 124.65, 126.82, 133.49, 151.30, 166.56, 169.29 and 174.78 (Ar-C and CO); MS (EI): *m*/*z* (%) 249 (M^+^, 100), 250 (M^+^+1, 14.6). Anal. calcd. for C_10_H_7_N_3_OS_2_ (249.31): C, 48.18; H, 2.83; N, 16.85; S, 25.72. Found: C, 48.09; H, 2.94; N, 16.94; S, 25.65. Crystallographic Analysis for **3c**: The crystals were mounted on a glass fiber. All measurements were performed on a Rigaku R-AXIS RAPID diffractometer using filtered Mo-Kα radiation. The data were collected at a temperature of 20 ± 1 °C to a maximum 2θ value of 55.0° using the ω scanning technique. The structure was solved by charge flipping method and expanded using Fourier techniques. The non-hydrogen atoms were refined anisotropically. Hydrogen atoms were refined using the riding model. Crystal Data: C_10_H_7_N_3_OS_2_, M = 249.31, monoclinic, a = 18.289(2) Å, b = 5.0485(1) Å, c = 11.5483(4) Å, V = 1066.30(9) Å^3^, α = γ = 90.00°, β = 90.077(6)°, space group: P2_1_/c, Z = 4, D_calc_ = 1.553 g cm^−3^. No. of reflections measured 2444, 2θ_max_ = 54.9°, R1 = 0.0305. Figure 3 illustrates the structure as determined. Full data can be obtained on request from the CCDC [30].

*(Z)-Ethyl-4-(4-oxothiazolidin-2-ylideneamino)benzoate* (**3d**). Recrystallized from EtOH as pale yellow crystals, yield: 80%, m.p. 186–187 °C; IR (KBr): 𝑣/cm^−1^ 3200 (NH), 1719, 1672 (2CO); ^1^H-NMR (DMSO-*d_6_*): δ = 1.31 (t, *J* = 7.2 Hz, 3H, CH_3_), 4.03 (s, 2H, CH_2_), 4.29 (q, *J* = 7.2 Hz, 2H, CH_2_), 7.06 (d, *J* = 7.8 Hz, 1H, Ar-H), 7.85 (d, *J* = 7.8 Hz, 1H, Ar-H), 7.96 (d, *J* = 7.8 Hz, 2H, Ar-H) and 11.54 ppm (br, 1H, NH); ^13^C-NMR (DMSO-*d_6_*): δ = 14.20 (CH_3_), 34.42 (CH_2_), 60.58 (CH_2_), 119.71, 121.31, 125.51, 130.48, 142.80, 157.92, 165.72 and 174.33 (Ar-C and CO); MS (EI): *m*/*z* (%) 264 (M^+^, 100), 265 (M^+^+1, 17.80). Anal. calcd. For C_12_H_12_N_2_O_3_S (264.31): C, 54.53; H, 4.58; N, 10.60; S, 12.13; Found: 54.64; H, 4.49; N, 10.67; S, 12.06. Crystallographic Analysis for **3d**. The crystals were mounted on a glass fiber. All measurements were performed on Bruker X8 Prospector. The data were collected at a temperature of 20 ± 1 °C to a maximum θ value of 66.61° using the ω scanning technique. The structure was solved by direct method using SHELXS-97 (Sheldrick, 2008) and refined by Full-matrix least-squares on F^2^. The non-hydrogen atoms were refined anisotropically. Data were corrected for absorption effects using the multi-scan method (SADABS). Crystal Data. C_12_H_12_N_2_O_3_S, M = 264.31, triclinic, a = 4.0829(3) Å, b = 5.5436(4) Å, c = 26.974(2) Å, V = 605.71(8) Å^3^, α = 84.625(6)°, β = 89.051(6)°, γ = 85.220(6)°, space group: P-1, Z = 2, D_calc_ = 1.449 Mg cm^−3^, No. of reflections measured 5850, θ_max_ = 66.0°, R1 = 0.0623. Figure 4 shows the structure as determined. Full data can be obtained on request from the CCDC [31].

### 3.4. General Procedure for the Synthesis of 2-Arylimino-5-arylidene-4-thiazolidinones ***7a–p***

*Method A*: Independent mixtures of 2-(heteroaryl or arylimino)thiazolidin-4-ones **3a–d** (5 mmol) and the appropriate arylidene malononitrile **5** (0.77 g, 5 mmol) in ethanol (30 mL) containing few drops of piperidine (5 drops) were stirred at reflux for 3 h. Then, the reaction mixtures were cooled to room temperature. The solid which formed was collected by filtration, washed with hot ethanol, and recrystallized from the appropriate solvent to afford **7a–p** respectively, as pure substances.

*Method B*: A mixture of 2-(heteroaryl or arylimino)thiazolidin-4-ones **3a–d** (5 mmol) and the appropriate arylaldehyde (5 mmol) in acetic acid (25 mL) containing sodium acetate (10 mmol) was refluxed for 5 h. The reaction mixture was then cooled to room temperature and poured into ice-cold water. The precipitate was filtered off and washed with water and the resulting crude product was purified by recrystallization from the appropriate solvent.

*(2Z,5Z)-5-Benzylidene-2-(2-chloro-5-nitrophenylimino)thiazolidin-4-one* (**7a**). Recrystallized from dioxane/DMF (1:2) mixture as creamy crystals; yield: (A: 84%, B: 71%), m.p. 240–241 °C; IR (KBr): 𝑣/cm^−1^ 3121 (NH), 1729 (CO); ^1^H-NMR (DMSO-*d_6_*): δ = 7.42–7.49 (m, 5H, Ar-H), 7.71 (s, 1H, olefinic CH), 7.82 (d, *J* = 7.6 Hz, 1H, Ar-H), 8.02–8.05 (m, 2H, Ar-H) and 12.86 ppm (s, 1H, NH); ^13^C-NMR (DMSO-*d_6_*): δ = 117.28, 120.51, 122.10, 129.25, 129.80, 130.15, 130.62, 131.18, 133.01, 146.07, 146.93, 154.28, 160.98 and 167.22 ppm (Ar-C and CO); MS (EI): *m*/*z* (%) 359 (M^+^, 82.3), 360 (M^+^+1, 20.22). Anal. calcd. for C_16_H_10_ClN_3_O_3_S (359.79): C, 53.41; H, 2.80; N, 11.68; S, 8.91. Found: C, 53.34; H, 2.68; N, 11.75; S, 8.84.

*(2Z,5Z)-2-(2-Chloro-5-nitrophenylimino)-5-(4-methylbenzylidene)thiazolidin-4-one* (**7b**). Obtained from dioxane/DMF (1:1) mixture as yellowish white crystals; yield: (A: 83%, B: 68%), m.p. 250–251 °C; IR (KBr): 𝑣/cm^−1^ 3132 (NH), 1731 (CO); ^1^H-NMR (DMSO-*d_6_*): δ = 3.32 (s, 3H, CH_3_), 7.27 (d, *J* = 8.0 Hz, 2H, Ar-H), 7.39 (d, *J* = 8.0 Hz, 2H, Ar-H), 7.68 (s, 1H, olefinic CH), 7.84 (d, *J* = 7.6 Hz, 1H, Ar-H), 8.02–8.04 (m, 2H, Ar-H) and 12.82 ppm (s, 1H, NH); ^13^C-NMR (DMSO-*d_6_*): δ = 21.03(CH_3_), 117.25, 120.43, 120.81, 129.84, 130.20, 130.69, 131.15, 133.01,140.36, 146.10, 146.89, 154.28, 160.84 and 167.25 ppm (Ar-C and CO); MS (EI): *m*/*z* (%) 373 (M^+^, 71.75), 374 (M^+^+1, 18.60). Anal. calcd. for C_17_H_12_ClN_3_O_3_S (373.82): C, 54.62; H, 3.24; N, 11.24; S, 8.58. Found: C, 54.70; H, 3.17; N, 11.31; S, 8.66.

*(2Z,5Z)-2-(2-Chloro-5-nitrophenylimino)-5-(4-methoxybenzylidene)thiazolidin-4-one* (**7c**). Obtained from DMSO as yellow crystals; yield: (A: 77%, B: 65%), m.p. 247–248 °C; IR (KBr): 𝑣/cm^−1^ 3136 (NH), 1726 (CO); ^1^H-NMR (DMSO-*d_6_*): δ = 3.78 (s, 3H, OCH_3_), 7.03 (d, *J* = 8.4 Hz, 2H, Ar-H), 7.49 (d, *J* = 8.4 Hz, 2H, Ar-H), 7.69 (s, 1H, olefinic CH), 7.87 (d, *J* = 8.0 Hz, 1H, Ar-H), 8.04–8.10 (m, 2H, Ar-H) and 12.77 ppm (s, 1H, NH); ^13^C-NMR (DMSO-*d_6_*): δ = 55.39 (CH_3_), 114.83, 117.32, 118.88, 120.42, 125.47, 130.65, 131.18, 131.84, 133.06, 146.24, 146.93, 154.46, 160.75 and 167.38 ppm (Ar-C and CO); MS (EI): *m*/*z* (%) 389 (M^+^, 66.25), 390 (M^+^+1, 13.94). Anal. calcd. for C_17_H_12_ClN_3_O_4_S (389.82): C, 52.38; H, 3.10; N, 10.78; S, 8.23. Found: C, 52.43; H, 3.03; N, 10.84; S, 8.17. Crystallographic Analysis for **7c**: The crystals were mounted on a glass fiber. All measurements were performed on a Rigaku R-AXIS RAPID diffractometer using filtered Mo-Kα radiation. The data were collected at a temperature of 20 ± 1 °C to a maximum 2θ value of 55.0° using the ω scanning technique. The structure was solved by charge flipping method and expanded using Fourier techniques. The non-hydrogen atoms were refined anisotropically. Hydrogen atoms were refined using the riding model. Crystal Data: C_17_H_12_ClN_3_O_4_S, M = 389.82, monoclinic, a = 21.540(3) Å, b = 8.1718(9) Å, c = 25.489(3) Å, V = 4170.4(8) Å^3^, α = γ = 90.00°, β = 111.639(8)°, space group: P2_1_/c, Z = 8, D_calc_ = 1.490 g cm^−3^, No. of reflections measured 6925, 2θ_max_ = 50.0°, R1 = 0.0470. Figure 5 illustrates the structure as determined. Full data can be obtained on request from the CCDC [34].

*(2Z,5Z)-2-(2-Chloro-5-nitrophenylimino)-5-(4-nitrobenzylidene)thiazolidin-4-one* (**7d**). Recrystallized from dioxane/DMF (1:2) mixture as yellow crystals; yield: (A: 73%, B: 59%), m.p. 263–264 °C; IR (KBr): 𝑣/cm^−1^ 3292 (NH), 1733 (CO); ^1^H-NMR (DMSO-*d_6_*): δ = 7.78 (d, *J* = 8.4 Hz, 2H, Ar-H), 7.83 (s, 1H, olefinic CH), 7.88 (d, *J* = 8.0 Hz, 1H, Ar-H), 8.06–8.08 (m, 2H, Ar-H), 8.27 (d, *J* = 8.4 Hz, 2H, Ar-H) and 13.07 ppm (s, 1H, NH); ^13^C-NMR (DMSO-*d_6_*): δ = 117.27, 120.69, 124.22, 126.62, 128.02, 130.76, 131.26, 132.99, 139.32; 145.88, 146.93, 147.24, 153.67 and 166.87 ppm (Ar-C and CO); MS (EI): *m*/*z* (%) 404 (M^+^, 55.30), 405 (M^+^+1, 14.62). Anal. calcd. For C_16_H_9_ClN_4_O_5_S (404.79): C, 47.48; H, 2.24; N, 13.84; S, 7.92. Found: C, 47.56; H, 2.27; N, 13.91; S, 7.86.

*(2Z,5Z)-5-(4-Chlorobenzylidene)-2-(2-chloro-5-nitrophenylimino)thiazolidin-4-one* (**7e**). Recrystallized from DMF as yellowish white crystals; yield: (A: 75%, B: 66%), m.p. 253–254 °C; IR (KBr): 𝑣/cm^−1^ 3128 (NH), 1724 (CO); ^1^H-NMR (DMSO-*d_6_*): δ = 7.51–7.52 (m, 4H, Ar-H), 7.71 (s, 1H, olefinic CH), 7.84 (d, *J* = 8.0 Hz, 1H, Ar-H), 8.03–8.05 (m, 2H, Ar-H) and 12.89 ppm (s, 1H, NH); MS (EI): *m*/*z* (%) 393 (M^+^, 45.30), 394 (M^+^+1, 12.95). Anal. calcd. for C_16_H_9_Cl_2_N_3_O_3_S (394.24): C, 48.75; H, 2.30; N, 10.66; S, 8.13. Found: C, 48.82; H, 2.23; N, 10.58; S, 8.19.

*(2Z,5Z)-2-(2-Chloro-5-nitrophenylimino)-5-(thiophen-2-ylmethylidene)thiazolidin-4-one* (**7f**). Obtained from dioxane as pale brown crystals; yield: (A: 81%, B: 70%), m.p. 266–267 °C; IR (KBr): 𝑣/cm^−1^ 3112 (NH), 1727 (CO); ^1^H-NMR (DMSO-*d_6_*): δ = 7.24 (t, *J* = 4.8 Hz, 1H, Ar-H), 7.62 (d, *J* = 4.8 Hz, 1H, Ar-H), 7.86 (s, 1H, olefinic CH), 7.90 (d, *J* = 4.8 Hz, 1H, Ar-H), 8.00 (s, 1H, Ar-H), 8.05–8.07 (m, 2H, Ar-H) and 12.83 ppm (s, 1H, NH); ^13^C-NMR (DMSO-*d_6_*): δ = 117.26, 119.64, 120.51, 123.98, 128.85, 131.18, 132.45, 133.10, 133.94, 137.16, 146.10, 146.88, 153.71 and 166.94 ppm (Ar-C and CO); MS (EI): *m*/*z* (%) 365 (M^+^, 86.77), 366 (M^+^+1, 23.48). Anal. calcd. For C_14_H_8_ClN_3_O_3_S_2_ (365.82): C, 45.97; H, 2.20; N, 11.49; S, 17.53. Found: C, 46.05; H, 2.26; N, 11.43; S, 17.44.

*(2Z,5Z)-Ethyl-4-(5-benzylidene-4-oxothiazolidin-2-ylideneamino)benzoate* (**7g**). Recrystallized from DMF as yellow crystals; yield: (A: 74%, B: 69%), m.p. 291–292 °C; IR (KBr): 𝑣/cm^−1^ 3199 (NH), 1709, 1678 (2CO); ^1^H-NMR (DMSO-*d_6_*): δ = 1.33 (t, *J* = 6.8 Hz, 3H, CH_3_), 4.32 (q, *J* = 6.8 Hz, 2H, CH_2_), 7.15 (d, *J* = 7.2 Hz, 2H, Ar-H), 7.47–8.00 (m, 8H, Ar-H and olefinic CH) and 12.34 ppm (br, 1H, NH); MS (EI): *m*/*z* (%) 352 (M^+^, 19.50), 353 (M^+^+1, 5.20). Anal. calcd. for C_19_H_16_N_2_O_3_S (352.41): C, 64.76; H, 4.58; N, 7.95; S, 9.10; Found: C, 64.84; H, 4.65; N, 8.01; S, 9.18.

*(2Z,5Z)-2-(4-Acetylphenylimino)-5-benzylidenethiazolidin-4-one* (**7h**). Recrystallized from DMF as pale brown crystals; yield: (A: 90%, B: 79%), m.p. above 300 °C; IR (KBr): 𝑣/cm^−1^ 3199 (NH), 1711, 1676 (CO); ^1^H-NMR (DMSO-*d_6_* at 110 °C): δ = 2.58 (s, 3H, CH_3_), 7.41–7.43 (m, 3H, Ar-H), 7.47–7.56 (m, 4H, Ar-H), 7.69 (s, 1H, olefinic CH), 8.00 (d, *J* = 8.0 Hz, 2H, Ar-H) and 12.34 ppm (s, 1H, NH); MS (EI): *m*/*z* (%) 322 (M^+^, 15.45), 323 (M^+^+1, 4.15). Anal. calcd. for C_18_H_14_N_2_O_2_S (322.39): C, 67.06; H, 4.38; N, 8.69; S, 9.95. Found: C, 66.98; H, 4.51; N, 8.63; S, 10.03.

*(2Z,5Z)-2-(4-Acetylphenylimino)-5-(4-methylbenzylidene)thiazolidin-4-one* (**7i**). Recrystallized from DMF as pale brown crystals; yield: (A: 93%, B: 81%), m.p. above 300 °C; IR (KBr): 𝑣/cm^−1^ 3196 (NH), 1710, 1674 (2CO); ^1^H-NMR (DMSO-*d_6_* at 110 °C): δ = 2.35 (s, 3H, CH_3_), 2.56 (s, 3H, CH_3_), 7.30 (d, *J* = 7.6 Hz, 2H, Ar-H), 7.43–7.45 (m, 4H, Ar-H), 7.65 (s, 1H, olefinic CH), 7.98 (d, *J* = 8.0 Hz, 2H, Ar-H) and 11.84 ppm (s, 1H, NH); MS (EI): *m*/*z* (%) 336 (M^+^, 55.0), 337 (M^+^+1, 13.50). Anal. calcd. for C_19_H_16_N_2_O_2_S (336.42): C, 67.84; H, 4.79; N, 8.33; S, 9.53. Found: C, 67.91; H, 4.75; N, 8.39; S, 9.45.

*(2Z,5Z)-2-(4-Acetylphenylimino)-5-(4-methoxybenzylidene)thiazolidin-4-one* (**7j**). Recrystallized from DMF as yellow crystals; yield: (A: 89%, B: 75%), m.p. above 300 °C; IR (KBr): 𝑣/cm^−1^ 3192 (NH), 1713, 1672 (2CO); ^1^H-NMR (DMSO-*d_6_* at 110 °C): δ = 2.56 (s, 3H, CH_3_), 3.82 (s, 3H, OCH_3_), 7.04 (d, *J* = 8.4 Hz, 2H, Ar-H), 7.39–7.49 (m, 4H, Ar-H), 7.64 (s, 1H, olefinic CH), 7.98 (d, *J* = 8.0 Hz, 2H, Ar-H) and 11.81 ppm (s, 1H, NH); ^13^C-NMR (DMSO-*d_6_* at 110 °C): δ = 26.68 (CH_3_), 55.96 (CH_3_), 115.09, 115.43, 1121.44, 126.75, 130.03, 130.26, 130.76, 131.91, 134.11, 143.95, 161.31, 166.74 and 196.84 ppm (Ar-C and CO); MS (EI): *m*/*z* (%) 352 (M^+^, 46.20), 353 (M^+^+1, 12.15). Anal. calcd. for C_19_H_16_N_2_O_3_S (352.41): C, 64.76; H, 4.58; N, 7.95; S, 9.10. Found: C, 64.79; H, 4.65; N, 7.86; S, 8.99.

*(2Z,5Z)-2-(4-Acetylphenylimino)-5-(4-nitrobenzylidene)thiazolidin-4-one* (**7k**). Recrystallized from DMF as yellow crystals; yield: (A: 92%, B: 84%), m.p. above 300 °C; IR (KBr): 𝑣/cm^−1^ 3198 (NH), 1718, 1674 (2CO); ^1^H-NMR (DMSO-*d_6_* at 110 °C): δ = 2.57 (s, 3H, CH_3_), 7.48–7.78 (m, 4H, Ar-H), 7.98 (s, 1H, olefinic CH), 8.10–8.27 (m, 4H, Ar-H) and 12.09 ppm (s, 1H, NH); MS (EI): *m*/*z* (%) 367 (M^+^, 100), 368 (M^+^+1, 23.80). Anal. calcd. for C_18_H_13_N_3_O_4_S (367.39): C, 58.85; H, 3.57; N, 11.44; S, 8.73. Found: C, 58.77; H, 3.66; N, 11.48; S, 8.79.

*(2Z,5Z)-2-(4-Acetylphenylimino)-5-(4-chlorobenzylidene)thiazolidin-4-one* (**7l)**. Recrystallized from DMF as pale brown crystals; yield: Yield: (A: 95%, B: 86%), m.p. above 300 °C; IR (KBr): 𝑣/cm^−1^ 3196 (NH), 1715, 1678 (2CO); ^1^H-NMR (DMSO-*d_6_* at 110 °C): δ = 2.56 (s, 3H, CH_3_), 7.40–7.54 (m, 6H, Ar-H), 7.67 (s, 1H, olefinic CH), 7.98 (d, *J* = 8.4 Hz, 2H, Ar-H) and 11.94 ppm (s, 1H, NH); MS (EI): *m*/*z* (%) 356 (M^+^, 78.50), 357 (M^+^+1, 19.95). Anal. calcd. for C_18_H_13_ClN_2_O_2_S (356.83): C, 60.59; H, 3.67; N, 7.85; S, 8.99. Found: C, 60.67; H, 3.64; N, 7.88; S, 8.93.

*(2Z,5Z)-2-(4-Acetylphenylimino)-5-(thiophen-2-ylmethylidene)thiazolidin-4-one* (**7m**). Recrystallized from DMF as yellow crystals; yield: (A: 87%, B: 78%), m.p. above 300 °C; IR (KBr): 𝑣/cm^−1^ 3195 (NH), 1716, 1672 (2CO); ^1^H-NMR (DMSO-*d_6_* at 110 °C): δ = 2.58 (s, 3H, CH_3_), 7.20–8.00 (m, 8H, Ar-H and olefinic CH) and 11.95 ppm (s, 1H, NH); MS (EI): *m*/*z* (%) 328 (M^+^, 49.55), 329 (M^+^+1, 10.25). Anal. calcd. for C_16_H_12_N_2_O_2_S_2_ (328.41): C, 58.52; H, 3.68; N, 8.53; S, 19.53. Found: C, 58.59; H, 3.76; N, 8.56; S, 19.44.

*(2Z,5Z)-2-(Benzothiazol-2-ylimino)-5-(4-methylbenzylidene)thiazolidin-4-one* (**7n**). Recrystallized from dioxane as yellow crystals; yield: (A: 86%, B: 73%), m.p. 247–248 °C; IR (KBr): 𝑣/cm^−1^ 3125 (NH), 1696 (CO); ^1^H-NMR (DMSO-*d_6_*): δ = 2.37 (s, 3H, CH_3_), 7.35–7.39 (m, 3H, Ar-H), 7.50 (t, *J* = 7.6 Hz, 1H, Ar-H), 7.58 (d, *J* = 8.0 Hz, 2H, Ar-H), 7.74 (s, 1H, olefinic CH), 7.93 (d, *J* = 7.6 Hz, 1H, Ar-H), 7.99 (d, *J* = 7.6 Hz, 1H, Ar-H) and 12.88 ppm (s, 1H, NH); MS (EI): *m*/*z* (%) 351 (M^+^, 52.6), 352 (M^+^+1,12.2). Anal. calcd. for C_18_H_13_N_3_OS_2_ (351.45): C, 61.52; H, 3.73; N, 11.96; S, 18.25 Found: C, 61.45; H, 3.81; N, 12.02; S, 18.32.

*(2Z,5Z)-2-(Benzothiazol-2-ylimino)-5-(thiophen-2-ylmethylidene)thiazolidin-4-one* (**7o**). Obtained from dioxane as yellow crystals; yield: (A: 86%, B: 79%), m.p. 276–277 °C; IR (KBr): 𝑣/cm^−1^ 3136 (NH), 1692 (CO); ^1^H-NMR (DMSO-*d_6_*): δ = 7.33 (t, *J* = 5.4 Hz, 1H, Ar-H), 7.39 (t, *J* = 7.6 Hz, 1H, Ar-H), 7.52 (t, *J* = 7.6 Hz, 1H, Ar-H), 7.73 (s, 1H, olefinic CH), 7.91 (d, *J* = 7.6 Hz, 1H, Ar-H), 8.01 (d, *J* = 7.6 Hz, 1H, Ar-H), 8.06–8.10 (m, 2H, Ar-H) and 12.89 ppm (s, 1H, NH); ^13^C-NMR (DMSO-*d_6_*): δ = 121.67, 121.94, 122.12, 124.48, 125.81, 126.56, 129.00, 133.16, 133.43, 134.92, 137.38, 150.75, 158.06, 166.83 and 168.22, ppm (Ar-C and CO); MS (EI): *m*/*z* (%) 343 (M^+^, 80.5), 344 (M^+^+1, 15.7). Anal. calcd. for C_15_H_9_N_3_OS_3_ (343.45): C, 52.46; H, 2.64; N, 12.23; S, 28.01. Found: C, 52.38; H, 2.73; N, 12.31; S, 27.94.

*(2Z,5Z)-2-(Benzothiazol-2-ylimino)-5-[4-(dimethylamino)benzylidene]thiazolidin-4-one* (**7p**). Obtained from dioxane as orange crystals; yield: (A: 90%, B: 77%), m.p. 299–300 °C; IR (KBr): 𝑣/cm^−1^ 3129 (NH), 1711 (CO); ^1^H-NMR (DMSO-*d_6_*): δ = 3.04 (s, 6H, 2CH_3_), 6.87 (d, *J* = 8.4 Hz, 2H, Ar-H), 7.36 (t, *J* = 8.0 Hz, 1H, Ar-H), 7.50 (t, *J* = 8.0 Hz, 1H, Ar-H), 7.55 (d, *J* = 8.4 Hz, 2H, Ar-H), 7.66 (s, 1H, olefinic CH), 7.93 (d, *J* = 8.0 Hz, 1H, Ar-H), 8.00 (d, *J* = 8.0 Hz, 1H, Ar-H) and 12.68 ppm (s, 1H, NH); MS (EI): *m*/*z* (%) 380 (M^+^, 26.75), 381 (M^+^+1, 7.65). Anal. calcd. for C_19_H_16_N_4_OS_2_ (380.49): C, 59.98; H, 4.24; N, 14.72; S, 16.85. Found: C,60.07; H, 4.16; N, 14.65; S, 16.89.

### 3.5. General Procedure for the Synthesis of the Enamines ***8***

Mixture of **3a** or **3c** (5 mmol), *N,N*-dimethylformamide dimethylacetal (DMF-DMA) (0.6 g, 5 mmol) in dioxane (20 mL) were stirred at reflux for 12 h. The separated solid product obtained on standing at room temperature was collected by filtration, washed by EtOH and recrystallized from dioxane to afford the corresponding enamines **8** as orange crystal. 

*(2Z,5Z)-2-(2-Chloro-5-nitrophenylimino)-5-[(dimethylamino)methylidene]thiazolidin-4-one* (**8a**). Yield: 75%, m.p. 260–261 °C; IR (KBr): 𝑣/cm^−1^ 2383 (NH), 1708 (CO); ^1^H-NMR (DMSO-*d_6_*): δ = 3.01 (s, 6H, 2CH_3_), 7.49 (s, 1H, olefinic CH), 7.79 (d, *J* = 8.4 Hz, 1H, Ar-H), 7.89–795 (m, 2H, Ar-H), and 11.77 ppm (s, 1H, NH); ^13^C-NMR (DMSO-*d_6_*): δ = 42.22 (2CH_3_), 84.91, 117.90, 119.92, 131.56, 133.97, 144.76, 147.41, 147.81, 156.48 and 168.40 ppm (Ar-C and CO); MS (EI): *m*/*z* (%) 326 (M^+^, 57.25), 327 (M^+^+1, 11.50). Anal. calcd. for C_12_H_11_ClN_4_O_3_S (326.76): C, 44.11; H, 3.39; N, 17.15; S, 9.81. Found: C, 44.18; H, 3.32; N, 17.24; S, 9.90.

*(2Z,5Z)-2-(Benzothiazol-2-ylimino)-5-[(dimethylamino)methylidene]thiazolidin-4-one* (**8b**). Yield: 79%, m.p. 205–206 °C; IR (KBr): 𝑣/cm^−1^ 3133 (NH), 1670 (CO); ^1^H-NMR (DMSO-*d_6_*): δ = 3.18 (s, 6H, 2CH_3_), 7.25 (t, *J* = 7.6 Hz, 1H, Ar-H), 7.39 (t, *J* = 7.6 Hz, 1H, Ar-H), 7.63 (s, 1H, olefinic CH), 7.75 (d, *J* = 7.6 Hz, 1H, Ar-H), 7.88 (d, *J* = 7.6 Hz, 1H, Ar-H) and 12.07 ppm (s, 1H, NH); ^13^C-NMR (DMSO-*d_6_*): δ = 41.71 (2CH_3_), 87.52, 120.76, 121.69, 123.48, 126.03, 132.53, 146.55, 151.19, 160.35, 167.19 and 168.97 ppm (Ar-C and CO); MS (EI): *m*/*z* (%) 304 (M^+^, 76.40), 305 (M^+^+1, 15.30). Anal. calcd. for C_13_H_12_N_4_OS_2_ (304.39): C, 51.30; H, 3.97; N, 18.41; S, 21.07. Found: C, 51.38; H, 4.05; N, 18.48; S, 21.13. Crystallographic Analysis for **8b**: The crystals were mounted on a glass fiber. All measurements were performed on Bruker X8 Prospector. The data were collected at a temperature of 20 ± 1 °C to a maximum θ value of 66.61° using the ω scanning technique. The structure was solved by direct method using SHELXS-97 (Sheldrick, 2008) and refined by Full-matrix least-squares on F^2^. The non-hydrogen atoms were refined anisotropically. Data were corrected for absorption effects using the multi-scan method (SADABS). Crystal Data: C_13_H_12_N_4_OS_2_, M = 304.39, monoclinic, a = 7.9497(5) Å, b = 7.2661(5) Å, c = 24.1772(17) Å, V = 1384.30(16) Å^3^, α = γ = 90.00°, β = 97.597(3)°, space group: P 1 21/c 1, Z = 4, D_calc_ = 1.461 Mg cm^−3^, No. of reflection measured 7648, θ_max_ = 66.69°, R1 = 0.0317. Figure 6 illustrates the structure as determined. Full data can be obtained on request from the CCDC [35].

### 3.6. General Procedure for the Synthesis of the Enamines ***9***

Mixtureof **3a** or **3c** (5 mmol), *N,N*-dimethylformamide dimethylacetal (DMF-DMA) (1.2 g, 10 mmol) in toluene (30 mL) were stirred at reflux for 6 h. The separated solid product obtained on standing at room temperature was collected by filtration, washed by EtOH and recrystallized from EtOH/dioxane (1:2) mixture to afford the corresponding enamines **9** as orange crystal. 

*(2Z,5Z)-2-(2-Chloro-5-nitrophenylimino)-5-[(dimethylamino)methylidene]-3-methylthiazolidin-4-one* (**9a**). Yield: 72%, m.p. 199–200 °C; IR (KBr): 𝑣/cm^−1^ 1703 (CO); ^1^H-NMR (DMSO-*d_6_*): δ = 3.04 (s, 6H, 2CH_3_), 3.25 (s, 3H, CH_3_), 7.63 (s, 1H, olefinic CH), 7.81 (d, *J* = 8.4 Hz, 1H, Ar-H), 7.90 (s, 1H, Ar-H) and 7.95 ppm (d, *J* = 8.4 Hz, 1H, Ar-H); ^13^C-NMR (DMSO-*d_6_*): δ = 28.85 (CH_3_), 41.93 (2CH_3_), 82.92, 117.29, 119.58, 131.16, 133.46, 144.92, 146.94, 147.09, 155.87 and 166.40 ppm (Ar-C and CO); MS (EI): *m*/*z* (%) 340 (M^+^, 41.55), 341 (M^+^+1, 9.70). Anal. calcd. for C_13_H_13_ClN_4_O_3_S (340.79): C, 45.82; H, 3.85; N, 16.44; S, 9.41. Found: C, 45.91; H, 3.79; N, 16.37; S, 9.36.

*(2Z,5Z)-2-(Benzothiazol-2-ylimino)-5-[(dimethylamino)methylidene]-3-methylthiazolidin-4-one* (**9b**). Yield: 79%, m.p. 211–212 °C; IR (KBr): 𝑣/cm^−1^ 1684 (CO); ^1^H-NMR (DMSO-*d_6_*): δ = 3.20 (s, 6H, 2CH_3_), 3.23 (s, 3H, CH_3_), 7.27 (t, *J* = 7.6 Hz, 1H, Ar-H), 7.40 (t, *J* = 7.6 Hz, 1H, Ar-H), 7.64 (s, 1H, olefinic CH), 7.76 (d, *J* = 7.6 Hz, 1H, Ar-H) and 7.90 ppm (d, *J* = 7.6 Hz, 1H, Ar-H); ^13^C-NMR (DMSO-*d_6_*): δ = 29.44 (CH_3_), 42.61 (2CH_3_), 85.93, 120.96, 121.72, 123.51, 126.06, 132.76, 146.58, 151.13, 159.38, 165.92 and 168.71 ppm (Ar-C and CO); MS (EI): *m*/*z* (%) 318 (M^+^, 33.9), 319 (M^+^+1, 7.45). Anal. calcd. for C_14_H_14_N_4_OS_2_ (318.42): C, 52.81; H, 4.43; N, 17.60; S, 20.14. Found: C, 52.74; H, 4.49; N, 17.52; S, 20.25.

### 3.7. (2Z,5Z)-2-(4-Acetylphenylimino)-5-[(dimethylamino)methylidene]thiazolidin-4-one *(**10**)*

A mixture of **3b** (10 mmol), N,N-dimethylformamide dimethylacetal (DMF-DMA) (1.2 g, 10 mmol) in toluene (25 mL) was stirred at reflux for 5 h. The separated solid product obtained on standing at room temperature was collected by filtration, washed by EtOH and recrystallized from dioxane/DMF (1:1) mixture as reddish brown crystals. Yield: 86%, m.p. 299–300 °C; IR (KBr): 𝑣/cm^−1^ 3194, 1678, 1657 (2CO); ^1^H-NMR (DMSO-*d_6_*): δ = 2.55 (s, 3H, CO*CH_3_*), 3.07 (s, 6H, 2CH_3_), 7.23 (d, *J* = 8.4 Hz, 2H, Ar-H), 7.57 (s, 1H, olefinic CH), 7.95 (d, *J* = 8.4 Hz, 2H, Ar-H) and 11.13 ppm (s, 1H, NH); MS (EI): *m*/*z* (%) 289 (M^+^, 55.50), 290 (M^+^+1, 10.80). Anal. calcd. for C_14_H_15_N_3_O_2_S (289.36): C, 58.11; H, 5.23; N, 14.52; S, 11.08. Found: C, 58.24; H, 5.17; N, 14.43; S, 10.95.

### 3.8. (2Z,5Z)-5-[(Dimethylamino)methylidene]-3-methyl-2-{4-[(E)-4-methlypent-2-enoyl]phenylimino} thiazolidin-4-one *(**11**)*

A mixture of **3b** (10 mmol), *N,N*-dimethylformamide dimethylacetal (DMF-DMA) (3.6 g, 30 mmol) in toluene (75 mL) was stirred at reflux for 72 h. The separated solid product obtained on standing at room temperature was collected by filtration, washed with EtOH and recrystallized from EtOH/dioxane (2:1) mixture to afford the corresponding enaminone **11** as deep orange crystals. Yield: 81%, m.p. 236–238 °C; IR (KBr): 𝑣/cm^−1^ 1686, 1677 (2CO); ^1^H-NMR (DMSO-*d_6_*): δ = 2.91 (s, 3H, CH_3_), 3.02 (s, 6H, 2CH_3_), 3.14 (s, 3H, CH_3_), 3.21 (s, 3H, CH_3_), 5.85 (d, *J* = 12 Hz, 1H, olefinic *CH=*CH), 7.00 (d, *J* = 8.4 Hz, 2H, Ar-H), 7.55 (s, 1H, olefinic CH), 7.71 (d, *J* = 12 Hz, 1H, olefinic CH=*CH*), 7.90 (d, *J* = 8.4 Hz, 2H, Ar-H); ^13^C-NMR (DMSO-*d_6_*): δ = 28.87 (CH_3_), 37.14 (CH_3_), 41.80 (2CH_3_), 24.53 (CH_3_) 83.57, 90.71, 121.03, 128.72, 135.72, 144.14, 151.63, 152.80, 153.93, 166.65 and 184.84 ppm (Ar-C and CO); MS (EI): *m*/*z* (%) 358 (M^+^, 100), 359 (M^+^+1, 22.55). Anal. calcd. for C_18_H_22_N_4_O_2_S (358.47): C, 60.31; H, 6.19; N, 15.63; S, 8.94. Found: C, 60.25; H, 6.23; N, 15.57; S, 8.96. Crystallographic Analysis for **11**: The crystals were mounted on a glass fiber. All measurements were performed on a Rigaku R-AXIS RAPID diffractometer using filtered Mo-Kα radiation. The data were collected at a temperature of 20 ± 1 °C to a maximum 2θ value of 55.0° using the ω scanning technique. The structure was solved by charge flipping method and expanded using Fourier techniques. The non-hydrogen atoms were refined anisotropically. Hydrogen atoms were refined using the riding model. Crystal Data: C_18_H_22_N_4_O_2_S, M = 358.47, triclinic, a = 7.275(2) Å, b = 10.923(2) Å, c = 24.1772(17) Å, V = 887.0(3) Å^3^, α = 105.825(8)°, β = 99.679(7)°, γ = 95.624(7)°, space group: P-1 (#2), Z = 2, D_calc_ = 1.342 g cm^−3^, No. of reflections measured 3236, θ_max_ = 50.7°, R1 = 0.0558. Figure 7 illustrates the structure as determined. Full data can be obtained on request from the CCDC [36].

### 3.9. (2Z)-2-(Benzothiazol-2-ylimino)-5-(phenylhydrazono)thiazolidin-4-one *(**12**)*

A cold solution of benzenediazonium chloride (10 mmol) was prepared by adding a solution of sodium nitrite (1.4 g dissolved in 10 mL water) to cold solution of aniline hydrochloride (0.93 g of aniline in 10 mL, 6M HCl) with stirring. The resulting solution of benzenediazonium chloride was then added to a cold solution of 4-thiazolidinone **3c** (2.49 g, 10 mmol) in ethanol (100 mL) in the presence of sodium hydroxide (6.0 g, 15 mmol). The reaction mixture was stirred at room temperature for 1h and poured into ice cold water, the formed solid product was collected by filtration and washed with water then recrystallized from an EtOH to afford **12** as pale brown crystals, yield: 68%, m.p. 154–155 °C; IR (KBr): 𝑣/cm^−1^ 3250, 3125 (2NH), 1723 (CO); ^1^H-NMR (DMSO-*d_6_*): δ = 6.98 (t, *J* = 8.0 Hz, 1H, Ar-H), 7.33–7.41 (m, 4H, Ar-H), 7.46–7.53 (m, 2H, Ar-H), 7.98 (d, *J* = 8.0 Hz, 1H, Ar-H), 8.01 (d, *J* = 8.0 Hz, 1H, Ar-H), 11.01 (s, 1H, NH) and 12.67 ppm (s, 1H, NH); MS (EI): *m*/*z* (%) 353 (M^+^, 39.70), 354 (M^+^+1, 7.33). Anal. calcd. for C_16_H_11_N_5_OS_2_ (353.43): C, 54.38; H, 3.14; N, 19.82; S, 18.14. Found: C, 54.50; H, 3.25; N, 19.75; S, 18.20.

### 3.10. General Procedure for the Synthesis of Azolopyrimidines ***13–18***

Independent mixtures of **11** (1.075 g, 3 mmol) and the appropriate heteroaromatic amine (3 mmol) in pyridine (20 mL) were stirred at reflux for 24 h. The reaction mixtures were cooled to room temperature and poured into ice cold water then acidified with hydrochloric acid (2 N), forming solids that were collected by filtration and washed with water then MeOH and recrystallized from the indicated solvent.

*(2Z,5Z)-5-[1-(Dimethylamino)methylidene]-3-methyl-2-[4-(1,2,4-triazolo[1,5-a]pyrimidin-7-yl)phenyl-imino]thiazolidin-4-one* (**13**). Recrystallized from EtOH as yellow crystals, yield: 95%, m.p. 212–213 °C; IR (KBr): 𝑣/cm^−1^ 1701 (CO); ^1^H-NMR (DMSO-*d_6_*): δ = 3.05 (s, 6H, 2CH_3_), 3.24 (s, 3H, CH_3_), 7.22 (d, *J* = 8.4 Hz, 2H, Ar-H), 7.58 (s, 1H, olefinic CH), 7.66 (d, *J* = 4.8 Hz, 1H, pyrimidine *H*), 8.30 (d, *J* = 8.4 Hz, 2H, Ar-H), 8.75 (s, 1H, triazole *H*) and 8.92 ppm (d, *J* = 4.8 Hz, 1H, pyrimidine *H*); ^13^C-NMR (DMSO-*d_6_*): δ = 28.87 (CH_3_), 41.65 (2CH_3_), 83.26, 108.91, 121.66, 124.54, 131.03, 144.34, 146.92, 152.37, 153.27, 154.83, 155.42, 155.86 and 166.50 (Ar-C and CO); MS (EI): *m*/*z* (%) 379 (M^+^, 100), 380 (M^+^+1, 23.50). Anal. calcd. for C_18_H_17_N_7_OS (379.45): C, 56.98; H, 4.52; N, 25.84; S, 8.45. Found: C, 56.92; H, 4.44; N, 25.73; S, 8.54.

*(2Z,5Z)-5-[1-(Dimethylamino)methylidene]-3-methyl-2-[4-(2-phenylpyrazolo[1,5-a]pyrimidin-7-yl)- phenylimino]thiazolidin-4-one* (**14**). Recrystallized from dioxane as yellow crystals, yield: 92%, m.p. 251–252 °C; IR (KBr): 𝑣/cm^−1^ 1690 (CO); ^1^H-NMR (DMSO-*d_6_*): δ = 3.06 (s, 6H, 2CH_3_), 3.25 (s, 3H, CH_3_), 7.23 (d, *J* = 8.4 Hz, 2H, Ar-H), 7.30–7.33 (m, 2H, pyrimidine *H* and pyrazole H), 7.45 (t, *J* = 7.6 Hz, 1H, Ar-H), 7.51 (t, *J* = 7.6 Hz, 2H, Ar-H), 7.59 (s, 1H, olefinic CH), 8.07 (d, *J* = 7.6 Hz, 2H, Ar-H), 8.34 (d, *J* = 8.4 Hz, 2H, Ar-H) and 8.58 ppm (d, *J* = 4.8 Hz, 1H, pyrimidine *H*); ^13^C-NMR (DMSO-*d_6_* at 110 °C): δ = 29.23 (CH_3_), 42.28 (2CH_3_), 84.92, 93.65, 107.45, 121.93, 126.24, 126.85, 129.17, 129.29, 131.11, 133.34, 144.57, 145.56, 149.82, 151.57, 152.16, 153.31, 155.35 and 167.13 (Ar-C and CO); MS (EI): *m*/*z* (%) 454 (M^+^, 100), 455 (M^+^+1, 21.95). Anal. calcd. for C_25_H_22_N_6_OS (454.56): C, 66.06; H, 4.88; N, 18.49; S, 7.05. Found: C, 66.11; H, 4.91; N, 18.55; S, 7.12. Crystallographic Analysis for **14**: The crystals were mounted on a glass fiber. All measurements were performed on a Rigaku R-AXIS RAPID diffractometer using filtered Mo-Kα radiation. The data were collected at a temperature of 20 ± 1 °C to a maximum 2θ value of 55.0° using the ω scanning technique. The structure was solved by charge flipping method and expanded using Fourier techniques. The non-hydrogen atoms were refined anisotropically. Hydrogen atoms were refined using the riding model. Crystal Data: C_25_H_22_N_6_OS, M = 454.56, monoclinic, a = 7.6658(8) Å, b = 10.612(2) Å, c =29.279(4) Å, V = 2378.2(5) Å^3^, α = γ = 90.00°, β = 93.200(7)°, space group: P2_1_/c (#14), Z = 4, D_calc_ = 1.392 g cm^−3^, No. of reflection measured 4333, θ_max_ = 50.6°, R1 = 0.066. Figure 8 illustrates the structure as determined. Full data can be obtained on request from the CCDC [37].

*7-{4-[(Z)5-(1-Dimethylaminomethylidene)-3-methyl-4-oxo-thiazolidin-(2Z)-ylideneamino]phenyl}-N-(2-chloro-5-nitrophenyl)pyrazolo[1,5-a]pyrimidine-3-carboxamide * (**15**). Recrystallized from dioxane/ DMF (2:1) mixture as orange crystals, yield: 87%, m.p. 294–295 °C; IR (KBr): 𝑣/cm^−1^ 3246 (NH), 1697, 1680 (2CO); ^1^H-NMR (DMSO-*d_6_* at 110 °C): δ = 3.07 (s, 6H, 2CH_3_), 3.27 (s, 3H, CH_3_), 7.24 (d, *J* = 8.4 Hz, 2H, Ar-H), 7.55 (s, 1H, olefinic CH), 7.59 (d, *J* = 4.8 Hz, 1H, pyrimidine *H*), 7.86 (d, *J* = 7.8 Hz, 1H, Ar-H), 7.97 (d, *J* = 7.8 Hz, 1H, Ar-H), 8.29 (d, *J* = 8.4 Hz, 2H, Ar-H), 8.81 (s, 1H, pyrazole *H*), 8.94 (d, *J* = 4.8 Hz, 1H, pyrimidine *H*), 9.48 (s, 1H, Ar-H), and 10.86 ppm (s, 1H, NH); MS (EI): *m*/*z* (%) 576 (M^+^, 100), 577 (M^+^+1, 44.85). Anal. calcd. for C_26_H_21_ClN_8_O_4_S (577.03): C, 54.12; H, 3.67; N, 19.42; S, 5.56. Found: C, 54.19; H, 3.65; N, 19.38; S, 5.64.

*7-{4-[(Z)5-(1-Dimethylaminomethylidene)-3-methyl-4-oxo-thiazolidin-(2Z)-ylideneamino]phenyl}-N-(4,6-dimethylpyrimidin-2-yl)pyrazolo[1,5-a]pyrimidine-3-carboxamide * (**16**). Recrystallized from EtOH/ dioxane (1:1) mixture as orange crystals, yield: 93%, m.p. above 300 °C; IR (KBr): 𝑣/cm^−1^ 3277 (NH), 1688, 1676 (2CO); ^1^H-NMR (DMSO-*d_6_*): δ = 2.42 (s, 6H, 2CH_3_), 3.06 (s, 6H, 2CH_3_), 3.24 (s, 3H, CH_3_), 7.01 (s, 1H, 4,6-dimethylpyrimidin *H*), 7.23 (d, *J* = 8.4 Hz, 2H, Ar-H), 7.60 (s, 1H, olefinic CH), 7.62 (d, *J* = 4.8 Hz, 1H, pyrimidine *H*), 8.26 (d, *J* = 8.4 Hz, 2H, Ar-H), 8.79 (s, 1H, pyrazole *H*), 8.98 (d, *J* = 4.8 Hz, 1H, pyrimidine *H*) and 10.60 ppm (s, 1H, NH); ^13^C-NMR (DMSO-*d_6_*): δ = 23.49 (2CH_3_), 28.91 (CH_3_), 42.18 (2CH_3_), 83.31, 104.76, 109.01, 115.59, 121.59, 124.62, 131.40, 144.46, 146.17, 146.72, 147.36, 152.36, 152.43, 153.25, 157.07, 157.97, 166.53 and 167.84 (Ar-C and CO); MS (EI): *m*/*z* (%) 527 (M^+^, 94.80), 528 (M^+^+1, 30.15). Anal. calcd. for C_26_H_25_N_9_O_2_S (527.61): C, 59.19; H, 4.78; N, 23.89; S, 6.08. Found: C, 59.25; H, 4.71; N, 23.98; S, 6.15.

*(2Z,5Z)-2-[4-(Benzimidazo[1,2-a]pyrimidine-4-yl)phenylimino]-5-[1-(dimethylamino)methylidene]-3-methylthiazolidin-4-one* (**17**). Recrystallized from EtOH/ dioxane (1:1) mixture as yellow crystals, yield: 96%, m.p. 281–282 °C; IR (KBr): 𝑣/cm^−1^ 1692 (CO); ^1^H-NMR (DMSO-*d_6_*): δ = 3.09 (s, 6H, 2CH_3_), 3.27 (s, 3H, CH_3_), 6.69 (d, *J* = 8.0 Hz, 1H, Ar-H), 7.06–7.10 (m, 2H, 1Ar-H and pyrimidine *H*), 7.27 (d, *J* = 8.4 Hz, 2H, Ar-H), 7.48 (t, *J* = 8.0 Hz, 1H, Ar-H), 7.61 (s, 1H, olefinic CH), 7.71 (d, *J* = 8.4 Hz, 2H, Ar-H), 7.88 (d, *J* = 8.0 Hz, 1H, Ar-H) and 8.86 ppm (d, *J* = 4.8 Hz, 1H, pyrimidine *H*); ^13^C-NMR (DMSO-*d_6_*): δ = 28.93 (CH_3_), 42.25 (2CH_3_), 83.47, 108.24, 114.75, 119.58, 120.76, 122.31, 125.77, 127.06, 127.20, 129.75, 144.25, 144.35, 149.54, 151.36, 151.64, 153.51, 155.86 and 166.65 (Ar-C and CO); MS (EI): *m*/*z* (%) 428 (M^+^, 100), 429 (M^+^+1, 24.10). Anal. calcd. for C_23_H_20_N_6_OS (428.52): C, 64.47; H, 4.70; N, 19.61; S, 7.48. Found: C, 64.54; H, 4.69; N, 19.64; S, 7.52.

*2((E)-3-{4-[(Z)5-(1-Dimethylaminomethylidene)-3-methyl-4-oxo-thiazolidin-(2Z)-ylideneamino]phen- yl}-3-oxoprop-1-enylamino)-4-phenylthiophene-3-carbonitrile ***(18)**. Recrystallized from dioxane as orange crystals, yield: 81%, m.p. 270–271 °C; IR (KBr): 𝑣/cm^−1^ 3116 (NH), 2206 (CN), 1699, 1654 (2CO); ^1^H-NMR (DMSO-*d_6_*): δ = 3.04 (s, 6H, 2CH_3_), 3.22 (s, 3H, CH_3_), 6.43 (d, *J* = 8.8 Hz, 1H, olefinic *CH=*CH), 7.10 (t, *J* = 7.6 Hz, 2H, Ar-H), 7.31 (s, 1H, thiophene H), 7.43–7.53 (m, 3H, Ar-H), 7.58 (s, 1H, olefinic CH), 7.60–7.65 (m, 2H, Ar-H), 7.83 (d, *J* = 8.8 Hz, 1H, olefinic CH*=CH*), 7.90 (d, *J* = 7.6 Hz, 1H, Ar-H), 8.04 (d, *J* = 7.6 Hz, 1H, Ar-H) and 12.97 ppm (brd, 1H, NH); MS (EI): *m*/*z* (%) 513 (M^+^, 100), 514 (M^+^+1, 29.80). Anal. calcd. for C_27_H_23_N_5_O_2_S_2_ (513.64): C, 63.14; H, 4.51; N, 13.63; S, 12.48. Found: C, 63.21; H, 4.44; N, 13.57; S, 12.39.

### 3.11. (2Z,5Z)-2-(Benzothiazol-2-ylimino)-5-[(1H-1,2,4-triazol-3-ylamino)methylidene]thiazolidin-4-one *(**19**)*

A mixture of the enamine **8b** (1.25 g, 5 mmol) and 3-amino-1,2,4-triazol (0.42 g, 5 mmol) in acetic acid (20 mL) containing anhydrous sodium acetate (10 mmol) was refluxed for 24 h. The reaction mixture was then cooled to room temperature and then poured into ice-cold water. The precipitate was filtered off and washed with water and the resulting crude product was purified by recrystallization from dioxane as brown crystals, yield: 74%, m.p. above 300 °C; IR (KBr): 𝑣/cm^−1^ 3195, 3142, 3115 (3NH), 1683 (CO); ^1^H-NMR (DMSO-*d_6_*): δ = 7.32 (t, *J* = 7.6 Hz, 1H, Ar-H), 7.46 (t, *J* = 7.6 Hz, 1H, Ar-H), 7.75 (d, *J* = 7.6 Hz, 1H, Ar-H), 7.95 (d, *J* = 7.6 Hz, 1H, Ar-H), 7.63 (d, *J* = 10.8 Hz, 1H, *CH*NH), 8.44 (br d, 1H, triazole H), 11.03 (s, 1H, NH), 12.24 (s, 1H, NH) and 13.76 ppm (br, 1H, NH); ^13^C-NMR (DMSO-*d_6_*): δ = 96.37, 120.91, 121.98, 123.94, 126.38, 132.88, 135.05, 143.78, 151.19, 158.97, 160.10, 167.66 and 168.94 ppm (Ar-C and CO); MS (EI): *m*/*z* (%) 343 (M^+^, 7.90), 344 (M^+^+1, 2.08). Anal. calcd. for C_13_H_9_N_7_OS_2_ (343.39): C, 45.47; H, 2.64; N, 28.55; S, 18.67. Found: C, 45.53; H, 2.73; N, 28.62; S, 18.54.

## 4. Conclusions

The results of the study described above have led to the development of a simple approach for the synthesis of a novel class of fused azolopyrimidines and 2-arylimino-5-arylidene-4-thiazolidinones, substances with potentially interesting biological and medicinal properties. Furthermore, the observations made during this work showed that the reaction of 4-thiazolidinones with arylidene malononitrile afforded only the 2-arylimino-5-arylidene-4-thiazolidinones and not corresponding pyranothiazole. In addition a new class of enaminone derivatives was synthesized, which can be used as precursors for the synthesis of a variety of interesting heterocyclic compounds.

## Figures and Tables

**Table 1:** Selected bond lengths and bond angles for **3a**.

Bond	Bond length(Å)	Bond	Bond angle(°)
N2-C5	1.420	C5-N2-C7	118.30
N2-C7	1.281	N2-C7-N3	121.20
N3-C8	1.375	S1-C7-N2	126.70
N3-C7	1.362	S1-C7-N3	112.10
S1-C7	1.749	S1-C9-C8	108.20
S1-C9	1.803	C7-N3-C8	117.40
C8-C9	1.505	N3-C8-C9	110.70

**Table 2:** Selected bond lengths and bond angles for **3c**.

Bond	Bond length(Å)	Bond	Bond angle(°)
N2-C7	1.385	C7-N2-C8	120.17
N2-C8	1.282	N2-C8-N3	118.36
N3-C8	1.381	S2-C8-N2	130.73
N3-C10	1.358	S2-C8-N3	110.91
S2-C8	1.747	S2-C9-C10	107.64
S2-C9	1.815	C8-N3-C10	118.21
C9-C10	1.501	N3-C10-C9	111.27

**Table 3:** Selected bond lengths and bond angles for **8b**.

Bond	Bond length(Å)	Bond	Bond angle(°)
N1-C4	1.369	C5-N1-C4	117.48
N1-C5	1.296	N1-C5-N3	120.98
N3-C5	1.363	N1-C5-S2	128.88
N3-C10	1.380	N3-C5-S2	110.11
S2-C5	1.753	C10-C6-S2	110.70
S2-C6	1.773	C5-N3-C10	117.96
C6-C7	1.367	N3-C10-C6	110.06
N2-C7	1.326	C5-S2-C6	91.11
